# Household financial burden associated with healthcare for older people in Viet Nam: a cross-sectional survey

**DOI:** 10.1186/s12961-022-00913-3

**Published:** 2022-11-29

**Authors:** Nguyen Hoang Giang, Nguyen The Vinh, Hoang Thi Phuong, Nguyen Thi Thang, Tran Thi Mai Oanh

**Affiliations:** Health Strategy and Policy Institute, Lane 196, Ho Tung Mau Street, Mai Dich Ward, Cau Giay District, Hanoi, Viet Nam

**Keywords:** Financial burden, Catastrophic health expenditure, Financial distress, Out-of-pocket health expenditure, Older households, Viet Nam

## Abstract

**Background:**

Population ageing and the associated increase in the healthcare needs of older people are putting pressure on the healthcare system in Viet Nam. The country prioritizes healthcare for older people and has developed financial protection policies to mitigate financial hardship due to out-of-pocket health expenditures (OOPHEs) borne by their households. This study examines the level and determinants of the financial burden of OOPHE among households with people aged ≥ 60 years in Viet Nam.

**Methods:**

A cross-sectional household survey was conducted among a sample of 1536 older people living in 1477 households in three provinces representing the North, Central and South regions of Viet Nam during 2019–2020. The financial outcomes were catastrophic health expenditure (CHE), using WHO's definition, and financial distress due to OOPHE. Multivariate binary logistic regression analysis was employed to determine the factors associated with these outcomes.

**Results:**

OOPHE for older household members accounted for 86.3% of total household health expenditure. Of households with older people, 8.6% (127) faced CHE, and 12.2% (181) experienced financial distress due to OOPHE. Households were at a higher risk of incurring financial burdens related to health expenditures if they had fewer household members; included only older people; were in rural or remote, mountainous areas; and had older members with noncommunicable diseases. There was no significant association between health insurance coverage and financial burden. However, when older people sought tertiary care or private care, the possibility of a household facing CHE increased. Regardless of the type and level of care, health service utilization by older people results in a higher likelihood of a household encountering financial distress.

**Conclusions:**

This study reveals that OOPHE for older people can impose substantial financial burdens on households, leading them to face CHE and financial distress. This study provides evidence to justify reforming financial protection policies and introducing policy interventions targeted at better protecting older people and their households from the financial consequences of OOPHE. There is also the need to strengthen the grassroots health facilities to provide primary care closer to home at lower costs, particularly for the management of noncommunicable diseases.

**Supplementary Information:**

The online version contains supplementary material available at 10.1186/s12961-022-00913-3.

## Background

According to United Nations estimates, the number of people aged ≥ 60 years will soar from 901 million in 2015 to 2.1 billion (or 21% of the world’s population) by 2050 [1]. While increases in life expectancy are a positive population outcome, population ageing generates many challenges to productivity and economic growth and may threaten social security systems [2]. For health systems, an increasing population of older adults might negatively affect progress towards providing universal health coverage, ensuring people’s access to essential health services and protecting them from financial hardship [3]. If health service coverage and financial assistance are inadequate, older persons are at higher risk of being exposed to catastrophic and impoverishing health expenditures. Because older people have greater and more diverse healthcare needs, they suffer the dual burden of deteriorating earnings and multiplying out-of-pocket health expenditures (OOPHEs) [4].

Viet Nam is a lower-middle-income country experiencing rapid population ageing. The percentage of the population ≥ 60 years of age will rise from 12.3% in 2020 to 26% in 2050 [5], which will also increase healthcare needs for the older population. A national study showed that about 70% of older people reported poor health status, and more than one third (37.4%) reported having had an acute illness or injury during the past year [6]. The study also found that a high proportion of older persons reported having a chronic noncommunicable disease (NCD), and on average, each older person suffered from three conditions [6]. Moreover, older people account for 88% of the burden of NCDs in Viet Nam [7]. Such statistics indicate that the older population has high healthcare needs, especially for comorbidities and chronic NCDs requiring long-term treatment, thus resulting in high costs for individuals, families and society.

To address the care needs of its ageing population, the government of Viet Nam prioritizes healthcare for older people through various laws and regulations. The health sector has implemented initiatives to improve their access to services and to protect people, including older adults, from financial hardship due to healthcare costs. According to the Law on Health Insurance, all people aged ≥ 80 years are entitled to a 100% subsidy of their health insurance premium. Health insurance coverage is also fully subsidized for those aged 60–79 years who live in poor households or belong to ethnic minority groups, while those living in near-poor households are entitled to a 70% discount on their health insurance premium. Pensioners who worked in the formal sector and retired at 60 years of age are entitled to free health insurance enrolment as part of their retirement benefits.

Viet Nam has successfully expanded health insurance coverage to more than 90% of its population, including, by 2018, nearly 95% of all older persons [8]. Despite this progress, OOPHE remains high, accounting for nearly 50% of total health expenditure in 2017 [9]. The high OOPHE is both a financial barrier, hampering people's access to healthcare, and the reason for the financial consequences faced by their households. In 2011, the Viet Nam National Ageing Survey found that about half of older persons with illness did not receive any treatment, and the most common reason was the lack of money to pay for services [6]. In 2018, a baseline survey of the nationally representative longitudinal study of aging in Viet Nam showed that 21.9% and 30% of older people used inpatient and outpatient care in the last 12 months. Additionally, about 13% of older people who were ill did not seek medical care, and lack of financial resources was the most prevalent reason [10].

Although some research has been conducted to determine the financial burden of health spending, there has been no detailed investigation in Viet Nam of the financial burden of health expenditure among households that include older people. A study using data from the Viet Nam Household Living Standards Survey (VHLSS), conducted every 2 years from 2002 to 2010, revealed that the proportion of households suffering from catastrophic health expenditures (CHEs) and impoverishment due to healthcare costs ranged from 3.9% to 5.7% and from 2.5% to 4.1%, respectively [11]. Despite the availability of these data, these indicators have not been assessed in relation to households with older persons, nor has there been an analysis of the extent to which older persons contribute to a high household burden of OOPHE and which types of services they received. Another study of older Vietnamese adults living in a rural area showed that older people with chronic conditions spent significantly more on healthcare than those without these diseases [12]. However, this study was carried out on a small scale and at the individual level. Furthermore, previous studies have not considered detailed components of out-of-pocket spending to understand how much older people have to pay for various healthcare services and the extent to which they have to rely on financial coping strategies, such as borrowing money from relatives or friends. To fill such gaps in the literature, this study aims to assess the level of the financial burden and identify factors associated with financial outcomes due to healthcare spending among households in Viet Nam that include older people.

## Methods

### Household survey

This cross-sectional household survey was conducted from November 2019 to August 2020 in three provinces representing Viet Nam's North, Central and South regions, namely Yen Bai, Thanh Hoa and Tien Giang.

A total of 1536 older people aged ≥ 60 years were selected using multistage cluster sampling. The study first divided districts into urban and rural strata in each province and then randomly chose one district from each stratum. In each district, two communes were randomly selected as sampling clusters; therefore, 12 communes were chosen in total. After the communes were determined, the lists of older people residing in each commune were gathered from the local provincial population centres (12 lists). From the list for each commune, older people were divided into three age groups (60–69, 70–79 and ≥ 80 years), which formed three separate sampling frames. As the predefined cluster size was 128 per commune, the number of participants in each age group within a cluster was randomly selected using proportionate random sampling. This means that the distribution of age groups in each cluster was identical to the actual age distribution in the corresponding commune.

As the sampling units are older people and randomly selected, there was a case where more than one eligible respondent lived in the same household. If the older people were not at home during the visit, the interviewer would return three times at most to reach the respondent. All older people were screened for cognitive function using the Mini-Cog tool before conducting the interview. No potential participant was absent or refused to take part in the study; however, 182 people were ineligible for inclusion because they were deaf or mute or had impaired cognitive function (a Mini-Cog score of ≤ 4 points) [13]. In these cases, new participants were chosen randomly from the list of unselected older persons to represent the same sex and age group. Therefore, the final sample was 1536 older people living in their own homes in 1477 households in the study sites during the survey.

### Data collection

Face-to-face interviews using a structured questionnaire were undertaken among the study participants. The key components of the questionnaire were adapted from two well-developed national surveys in Viet Nam, which warrants the validity and reliability of the measures. While the questions on households’ characteristics were based on the VHLSS 2018 [14], questions on health service utilization and health expenditures were adapted from a national presentation household survey conducted by the Health Strategy and Policy Institute in 2015 (findings of the surveys were partially published elsewhere [15]). It comprised questions to assess older people’s health status, healthcare utilization and out-of-pocket spending on their healthcare. In addition, the survey tool collected information about other household members’ demographic and socioeconomic characteristics, their health service use, health payments, household assets and housing conditions.

Local commune-based health workers were recruited to conduct the interviews. All interviewers attended a 2-day training workshop that included information about the questionnaire, interviewing skills and data confidentiality; the trainees also conducted some pilot interviews before the actual survey.

At the home visit, interviewers met directly with the eligible older people. Moreover, one adult household member was self-nominated as the most knowledgeable individual regarding the household’s general information and healthcare issues; in some cases, the older person also provided the general household information. To ensure data quality, members of the study team acted as field supervisors. The supervisors directly observed some of the first interviews and provided feedback. Random and unannounced spot-check visits were also undertaken.

### Healthcare setting

Viet Nam's health service delivery system uses a public–private mix in which the public sector takes the dominant role in service provision, especially for specialist and inpatient care. The public health system is divided into four levels that correspond to the country’s administrative divisions: commune, district, provincial and central. Each province has a provincial-level general hospital and several specialist hospitals. In each district, a district hospital and commune health stations (one per commune) provide primary healthcare services for the population in their catchment areas. At the community level, private health providers are well established, including polyclinics and specialist clinics, which provide outpatient services for local people. Local pharmacies are also readily available in the community, allowing people to purchase a broad range of medications, from over-the-counter to prescription medicines, including vitamins, painkillers, antibiotics and medications for chronic diseases, such as hypertension and diabetes.

Yen Bai is a mountainous province located in north-west Viet Nam, with a population of 0.82 million, of whom nearly 60% are ethnic minorities [16]. Apart from seven provincial public hospitals, the province has only one private hospital [17]. The second site, Thanh Hoa, has approximately 3.7 million residents, with 76% living in rural areas [16]. Eleven provincial public hospitals serve this north-central province, and healthcare in the private sector is well developed and includes 12 private hospitals [17]. Tien Giang province is situated in the Mekong Delta region of south-west Viet Nam. Approximately 1.7 million people, or 77% of the population, live in rural areas [16]. While the province has eight provincial hospitals in the public sector, only one private hospital was established in this province [17].

### Variables

#### Outcome variables

The study outcomes were the financial burden linked to OOPHE borne by households, including at least one person aged ≥ 60 years. The household-level financial outcomes were constructed using two indicators: CHE and financial distress. These measurements have been widely used to reflect the financial consequences of OOPHE by households [18–21].

In this study, household OOPHE was defined as direct spending on healthcare by individuals and households. The survey collected information on health expenditure for older people and all other household members, including an individual’s health expenses incurred at the time of services, such as health insurance co-payments, direct medical expenses (e.g. medications, laboratory tests, consultation fees), direct nonmedical expenses associated with accessing health services (e.g. food, transportation and accommodation) and other nonmedical expenses (e.g. informal gifts for health providers). OOPHE also encompasses home care for older members paid by households, self-medication and the purchase of medical devices. However, it does not include any prepayments for health services, for example, in the form of taxes or specific insurance premiums or contributions, and it is net of any reimbursements to individuals who made the payments where possible. The OOP spending on health services in the last 12 months was collected. While spending on inpatient care and health equipment was directly collected for the last 12 months, health expenses on self-medications and outpatients were collected for the last 4 weeks and then converted to 12-month figures.

CHE is defined as OOPHE for care that exceeds a certain threshold of household resources during a given period [22]. In this study, WHO’s approach was used to calculate CHE, which determines that a household faces CHE when its total OOPHE is ≥ 40% of its capacity to pay [23]. The household's capacity to pay was calculated by subtracting food expenditure from total household expenditures. Additionally, to ensure comparability with recent studies employing the financial protection outcomes of the Sustainable Development Goals (Indicator 3.8.2), we estimated the proportion of households with catastrophic spending on healthcare as a share of total household expenditure at 10% and 25% thresholds [24].

Financial distress due to spending on health was defined as financial activities or coping strategies employed by households to finance the cost of inpatient and outpatient services, for example, by borrowing money from friends or relatives, taking out loans from banks or other lenders, or selling assets. The outcome variable of financial distress was constructed as a binary variable, of which the variable is valued as “1” if a household does any coping methods (e.g. borrow, loan, selling assets) to finance the health expenses on any type of health services for the older people (outpatient, inpatient, home-based care) and “0” if a household did not mobilize external sources of money to cover health payments for their older members.

#### Covariates

Covariates were selected based on a review of the relevant literature and data availability. The independent variables were constructed as household-level data. These variables included the characteristics of the head of household (e.g. being aged ≥ 60 years, sex, ethnicity, educational level and occupational status) and the household (e.g. household size, the number of older people in the household, the presence of at least one child younger than 6 years), the composition of the household (older households: households with only older people versus multigenerational households where older people live with younger members), living in a rural area versus an urban area, and the province of residence. The variable of household health insurance status was also created with two categories: fully insured (e.g. all household members have health insurance) and not fully insured (at least one member does not have health insurance).

In this study, a wealth index was used as a proxy indicator of the household’s living standards. Following Filmer and Pritchett [25], a principal component analysis was undertaken to construct an asset score for each household according to household assets and durable products, the materials used for housing construction, the water source, type of toilet facility and ownership of agricultural land. The index was constructed separately for each area based on differing sets of household assets and amenities to reflect the relationship between living standards and properties in urban and rural areas. We then scaled these scores using the regression method guided by Rutstein [26]. Thus, a composite index enables comparability between urban and rural areas with the same level of wealth. The wealth index was then categorized into quintiles (Q1, Q2, Q3, Q4 and Q5). The poorest quintile (Q1) represents the 20% of households lowest on the wealth index, and the fifth quintile represents the 20% highest on the wealth index. Concerning the existence of NCDs among the older population, a household-level variable representing the number of NCD comorbidities suffered by key older household members was created with three groups (no NCDs: households where the key older respondent did not have any NCD; 1 NCD: households where the key older person had one NCD; 2+ NCDs: household with the key older person suffering two or more NCDs).

To account for the association between health spending and the level and type of care, we generated variables related to health service utilization by older people. The use of such variables is justified by prior studies in low- and middle-income countries indicating that certain types of care were significant predictors of health-related financial outcomes [15, 27–29]. These were created as continuous variables distinguished by the type of care (outpatient versus inpatient, public versus private) and type of health facility (using commune- or district-level care as primary healthcare and including tertiary care and private care). The variables were (i) outpatient care, defined as the total number of outpatient visits made by the key older members during the last month divided by the number of older members interviewed, and (ii) inpatient care, defined as the total number of inpatient admissions for the key older members during the past 12 months divided by the number of older household members interviewed.

### Statistical analysis

The unit of analysis was households with older adults. Concerning the missing data, less than 0.6% of data were missing for ethnicity, educational level and occupation of the household head; no data were missing on the outcome variables. All the analyses were based on complete cases. We used descriptive analytics for the socioeconomic characteristics of the households, healthcare utilization by older household members, the breakdown of OOPHE for older household members, and the household financial hardships experienced due to these expenditures. We analysed differences in CHE and financial coping strategies by household characteristics using the *t*-test, χ^2^ test and analysis of variance for statistical significance. We also used a multivariate logistic regression model for binary variables to explore determinant effects on CHE and the financial distress of households. Backward elimination was employed to identify the best set of predictors, as shown by descriptive analytics. Interaction and confounding effects were assessed in the model-building process; however, there was no significant interaction between covariates. The goodness of fit for the final regression models was examined using a likelihood ratio test, the Akaike information criterion and the Bayesian information criterion. The variance inflation factor was used to check multicollinearity, and a factor of < 10 was considered acceptable. A detailed explanation of the model-building process was presented in Additional file 1: Appendix 2. All analyses were performed using Stata 14 statistical software (StataCorp, College Station, TX, USA).

### Ethical approval

The scientific and ethical aspects of the study were reviewed and approved by the Institutional Scientific Research Committee of the Health Strategy and Policy Institute, Hanoi. Ethical approval was also obtained from the WHO Research Ethics Review Committee (protocol no. ERC.0003085). Local health authorities issued permission to conduct the field survey in their respective districts and provinces. All respondents were enrolled voluntarily and provided both verbal and written informed consent.

## Results

### Characteristics of the study households

Among the 1477 sampled households, there was a balanced distribution of urban and rural populations. The average household size was 3.2 persons, with 15.6% (231) of households including children younger than 6 years and 56.5% (835) including more than one person aged ≥ 60 years. In 42.2% (624) of households, people aged ≥ 60 years lived alone or only with a spouse. Moreover, 92.6% (1367) of household heads were aged ≥ 60 years, 53.1% (784) were men and 98.7% (1449) were members of the Kinh ethnic group. One third of heads of household had completed at least high school, and two thirds were not currently working.

In terms of healthcare, 79.4% (1173) of households had a key older respondents with one or more NCDs. The mean monthly outpatient visits per key older person at grassroots health facilities and tertiary facilities were 0.46 and 0.36, respectively. During the previous 12 months, the mean number of inpatient admissions of key older persons per household was higher at tertiary hospitals (0.19) than at district hospitals (0.09) and private health providers (0.02) (Table 1).

**Table 1:** Descriptive characteristics of households that included at least one member ≥ 60 years, Viet Nam

Variables	Total (*N* = 1477)	
	*n*	% or mean (SD)
**Household features**		
Urban versus rural		
Urban	720	48.7%
Rural	757	51.3%
Province		
Thanh Hoa	498	33.7%
Tien Giang	484	32.8%
Yen Bai	495	33.5%
No. of people in households (household size)	1477	3.2 (1.8)
Children < 6 years in the household		
No	1246	84.4%
Yes	231	15.6%
No. of people aged ≥ 60 years in the household		
1 person	642	43.5%
≥ 2 persons	835	56.5%
Household composition		
Multigenerational households	853	57.8%
Older households	624	42.2%
Health insurance status		
Not fully insured	174	11.8%
Fully insured	1303	88.2%
Wealth level (quintile)		
Poorest	296	20.0%
Poor	295	20.0%
Middle	296	20.0%
Rich	295	20.0%
Richest	295	20.0%
**Head of household**		
Aged ≥ 60 years		
No	110	7.4%
Yes	1367	92.6%
Sex		
Male	784	53.1%
Female	693	46.9%
Ethnicity		
Kinh	1449	98.7%
Ethnic minority	19	1.3%
Educational level		
Elementary or less	543	36.8%
Secondary school	419	28.4%
High school or higher	514	34.8%
Current occupational status		
Employed	490	33.3%
Pensioner	479	32.6%
Unemployed	502	34.1%
**Healthcare for key older members**		
Comorbidities		
No NCD	304	20.6%
1 NCD	551	37.3%
≥ 2 NCDs	622	42.1%
Mean (SD) no. outpatient visits (the last month)		
Primary healthcare facilities	1477	0.46 (0.86)
Tertiary health facilities	1477	0.36 (0.84)
Private health facilities	1477	0.13 (0.51)
Mean (SD) no. inpatient admissions (The last 12 months)		
District hospitals	1477	0.09 (0.43)
Tertiary hospitals	1477	0.19 (0.70)
Private hospitals	1477	0.02 (0.18)

*NCD* noncommunicable disease, *SD* standard deviation

### Incidence of CHE and financial distress

Table 2 shows the estimates of OOPHE borne by households that included at least one older person. On average, each household spent nearly 8 million Vietnamese dong (~US$ 350) on healthcare during the previous 12 months. OOPHE for older household members contributed most to total household health expenditure, accounting for 86.3%. Significantly, 8.6% (127) of households had OOPHE that exceeded 40% of their capacity to pay. When health spending for older household members was removed from total household health expenditures, the corresponding figure was only 1.96% (29). The descriptive data on households’ OOPHE for older people by type of health expenses is presented in Additional file 1: Appendix 1. Using definitions of CHE found in the Sustainable Development Goals, the percentage of households with OOPHE exceeding 10% of total household expenditure was 7.5%, and 1.8% exceeded 25% of total household expenditure.

**Table 2:** Financial burden borne by households that included people aged ≥ 60 years, Viet Nam

Out-of-pocket health expenditure	Total (*N* = 1477)
Mean (minimum–maximum) total household health expenditure in 1000 Vietnamese dong	7989 (0–108,280)
Mean (minimum–maximum) total household health expenditure for older people in 1000 Vietnamese dong	7378 (0–106,910)
Mean (minimum–maximum) total household health expenditure in 1000 Vietnamese dong (*estimated only for households that incurred health expenditure)*	10,591 (13–108,280)
Mean (minimum–maximum) total household health expenditure for older people in 1000 Vietnamese dong (*estimated only for households that incurred health expenditure for older people)*	9782 (13–106,910)
Mean percentage of health expenditure for older people as share of total household health expenditure (mean, SD)	86.3% (29.7)
% (no.) of households with health expenditure exceeding 40% of capacity to pay	8.6 (127)
% (no.) of households with health expenditure exceeding 40% of capacity to pay when health spending for older members is excluded	1.96 (29)
% (no.) of households with health expenditure exceeding 10% of total household expenditure	7.5 (111)
% (no.) of households with health expenditure exceeding 25% of total household expenditure	1.8 (26)
% (no.) of households facing financial distress due to spending for outpatient services	8.3 (122)
% (no.) of households facing financial distress due to spending for inpatient services	5.4 (79)
% (no.) of households facing financial distress due to spending for health services	12.3 (181)

The proportion of households suffering financial distress due to OOPHE was 12.3% (181). Households used different coping strategies to finance healthcare spending for older household members. Among the households suffering financial distress, 35.0% (63/181) of households had to borrow money from relatives or friends; 27.7% (50) got a loan from other individuals or agents, such as moneylenders; and 5.0% (9) sold properties. More than one third of households (43.9%) used their savings to pay for healthcare.

### Descriptive statistics

Table 3 shows the numbers and percentages of households experiencing CHE and financial distress against household-level covariates. Using the CHE threshold of 40% of nonfood expenditure, the highest incidence of CHE was seen among the households in Yen Bai province (16.6%; 82 households), followed by those in Thanh Hoa (7%; 35 households) and Tien Giang (1.9%; 9 households). CHE was more prevalent among households with fewer members (*P* < 0.001); without a child younger than 6 years; with only older people (18.9% versus 0.9% for others); with older members suffering from an NCD (8.9% for 1 NCD and 11.1% for ≥ 2 NCDs versus 2.6% of households without an older person with an NCD; *P* < 0.001); with heads aged ≥ 60 years who had completed at least secondary school (11.5% for secondary school and 9.5% for high school or higher; *P* = 0.002) or who were pensioners (11.1%; *P* = 0.037). Surprisingly, the incidence of CHE was higher in fully insured households than in households that were not fully insured (9.4% versus 2.3%; *P* = 0.002). Regarding health service utilization, households with CHE had older members with more outpatient visits at private health facilities and inpatient admissions to tertiary hospitals than households not incurring CHE.

**Table 3:** Households that include people ≥ 60 years of age with CHE and financial distress, by household covariates, Viet Nam^a^

Variable	CHE definition^b^					
	WHO		Sustainable Development Goals		Financial distress	
	Yes (*n* = 126)	No (*n* = 1351)	Yes (*n* = 111)	No (*n* = 1366)	Yes (*n* = 181)	No (*n* = 1296)
Household features						
Urban versus rural						
Urban	54 (7.5)	666 (92.5)	46 (6.4)	674 (93.6)	80 (11.1)	640 (88.9)
Rural	72 (9.5)	685 (90.5)	65 (8.6)	692 (91.4)	101 (13.3)	656 (86.7)
Province	***		***		*	
Thanh Hoa	35 (7.0)	463 (93.0)	38 (7.6)	460 (92.4)	51 (10.2)	447 (89.8)
Tien Giang	9 (1.9)	475 (98.1)	8 (1.7)	476 (98.3)	55 (11.4)	429 (88.6)
Yen Bai	82 (16.6)	413 (83.4)	65 (13.1)	430 (86.9)	75 (15.2)	420 (84.8)
Mean (SD) no. of people in household	1.6 (0.5)***	3.4 (1.9)	2.0 (1.1)***	3.3 (1.9)	3.0 (1.8)*	3.3 (1.9)
Children < 6 years	***		***			
No	126 (10.1)	1120 (89.9)	106 (8.5)	1140 (91.5)	157 (12.6)	1089 (87.4)
Yes	0 (0.0)	231 (100.0)	5 (2.2)	226 (97.8)	24 (10.4)	207 (89.6)
People aged ≥ 60 years in the household						
1 person	57 (8.9)	585 (91.1)	52 (8.1)	590 (91.9)	84 (13.1)	558 (86.9)
2+ persons	69 (8.3)	766 (91.7)	59 (7.1)	776 (92.9)	97 (11.6)	738 (88.4)
Household composition	***		***		*	
Multigenerational households	8 (0.9)	845 (99.1)	25 (2.9)	828 (97.1)	92 (10.8)	761 (89.2)
Older households	118 (18.9)	506 (81.1)	86 (13.8)	538 (86.2)	89 (14.3)	535 (85.7)
Health insurance status	***		***			
Not fully insured	4 (2.3)	170 (97.7)	3 (1.7)	171 (98.3)	19 (10.9)	155 (89.1)
Fully insured	122 (9.4)	1181 (90.6)	108 (8.3)	1195 (91.7)	162 (12.4)	1141 (87.6)
Wealth level (quintile)					*	
Poorest	22 (7.4)	274 (92.6)	14 (4.7)	282 (95.3)	44 (14.9)	252 (85.1)
Poor	29 (9.8)	266 (90.2)	25 (8.5)	270 (91.5)	44 (14.9)	251 (85.1)
Middle	36 (12.2)	260 (87.8)	32 (10.8)	264 (89.2)	40 (13.5)	256 (86.5)
Rich	18 (6.1)	277 (93.9)	19 (6.4)	276 (93.6)	25 (8.5)	270 (91.5)
Richest	21 (7.1)	274 (92.9)	21 (7.1)	274 (92.9)	28 (9.5)	267 (90.5)
Head of household						
Aged ≥ 60 years	*					
No	3 (2.7)	107 (97.3)	9 (8.2)	101 (91.8)	11 (10.0)	99 (90.0)
Yes	123 (9.0)	1,244 (91.0)	102 (7.5)	1,265 (92.5)	170 (12.4)	1,197 (87.6)
Sex						
Male	69 (8.8)	715 (91.2)	63 (8.0)	721 (92.0)	93 (11.9)	691 (88.1)
Female	57 (8.2)	636 (91.8)	48 (6.9)	645 (93.1)	88 (12.7)	605 (87.3)
Ethnicity			*			
Kinh	124 (8.6)	1,325 (91.4)	107 (7.4)	1,342 (92.6)	176 (12.1)	1,273 (87.9)
Ethnic minority	2 (10.5)	17 (89.5)	4 (21.1)	15 (78.9)	5 (26.3)	14 (73.7)
Educational level	**		***		*	
Elementary or less	29 (5.3)	514 (94.7)	17 (3.1)	526 (96.9)	76 (14.0)	467 (86.0)
Secondary school	48 (11.5)	371 (88.5)	50 (11.9)	369 (88.1)	59 (14.1)	360 (85.9)
High school or higher	49 (9.5)	465 (90.5)	44 (8.6)	470 (91.4)	46 (8.9)	468 (91.1)
Current occupational status	*		*		***	
Employed	33 (6.7)	457 (93.3)	34 (6.9)	456 (93.1)	51 (10.4)	439 (89.6)
Pensioner	53 (11.1)	426 (88.9)	47 (9.8)	432 (90.2)	39 (8.1)	440 (91.9)
Unemployed	38 (7.6)	464 (92.4)	28 (5.6)	474 (94.4)	88 (17.5)	414 (82.5)
Healthcare for key older members interviewed						
Comorbidities	***		***		***	
No NCD	8 (2.6)	296 (97.4)	6 (2.0)	298 (98.0)	6 (2.0)	298 (98.0)
1 NCD	49 (8.9)	502 (91.1)	36 (6.5)	515 (93.5)	66 (12.0)	485 (88.0)
≥ 2 NCDs	69 (11.1)	553 (88.9)	69 (11.1)	553 (88.9)	109 (17.5)	513 (82.5)
Mean (SD) no. outpatient visits in the last month						
Primary healthcare facilities	0.50 (0.80)	0.46 (0.86)	0.58 (1.17)	0.45 (0.82)	0.82 (1.06)***	0.41 (0.81)
Tertiary facilities	0.47 (0.98)	0.35 (0.83)	0.54 (1.06)*	0.35 (0.82)	0.54 (1.04)**	0.34 (0.81)
Private health facilities	0.23 (0.67)*	0.13 (0.49)	0.23 (0.68)*	0.13 (0.49)	0.23 (0.65)**	0.12 (0.48)
Mean (SD) no. inpatient admissions in the last 12 months						
District hospitals	0.14 (0.41)	0.09 (0.43)	0.19 (0.56)*	0.08 (0.41)	0.26 (0.62)***	0.07 (0.39)
Tertiary hospitals	0.34 (0.70)*	0.17 (0.70)	0.37 (0.78)**	0.17 (0.69)	0.48 (0.97)***	0.15 (0.64)
Private hospitals	0.04 (0.23)	0.02 (0.18)	0.07 (0.29)**	0.02 (0.17)	0.04 (0.19)	0.02 (0.18)

*CHE* catastrophic health expenditure, *NCD* noncommunicable disease, *SD* standard deviation, *P < 0.05; **P < 0.01; ***P < 0.001

^a^Values are number (%) unless otherwise indicated

^b^WHO defines CHE at a threshold of 40% of nonfood spending for a household, whereas Sustainable Development Goal indicator 3.8.2 defines it at a 10% threshold of total household expenditure

When CHE at a 10% threshold of total household expenditure was assessed, we observed the same patterns of bivariate associations as when WHO’s definition was used, except for healthcare service utilization by older household members. Compared with households not facing CHE, those experiencing CHE had older adults with significantly higher numbers of outpatient visits and inpatient admissions at all levels of care, except for outpatient visits to primary healthcare services.

In terms of coping with financial difficulties related to healthcare spending, 15.2% of households reported financial distress in Yen Bai, 11.4% in Tien Giang and 10.2% in Thanh Hoa. The incidence of financial distress was significantly higher among those in the lower wealth quintiles (Q1: 14.9%; Q2: 14.9%; Q3: 13.5%) than among those in the better-off groups (Q4: 8.5%; Q5: 9.5%). Notably, households that had older members with NCDs had higher rates of financial distress (1 NCD: 11.9% and > NCDs: 17.5%) than those without older members with NCDs (2%). Moreover, financial distress was significantly associated with higher utilization of all types of care except for inpatient admission to private health facilities.

### Determinants of financial burden due to health expenditure

The outputs of the base logistic regression models predicting the determinants CHE and financial distress among households with people aged ≥ 60 years are shown in Additional file 1: Appendix 3. Table 4 presents the results of the multivariate logistic regression models identifying the determinants of CHE and financial distress. The models indicate that household size, the presence of older members with NCDs, the province of residence and utilization of outpatient and inpatient services were significantly associated with CHE and financial distress resulting from OOPHE.

**Table 4:** Logistic regression models predicting the determinants of CHE and financial distress among households with people ≥ 60 years of age, Viet Nam

Variable	CHE (WHO’s definition) *N* = 1477		Financial distress *N* = 1471	
	Adjusted OR (95% CI)	*P*	Adjusted OR (95% CI)	*P*
Household size	0.18 (0.12–0.27)	0.000	0.89 (0.81–0.99)	0.024
Head of household’s occupational status	NA	NA		
Unemployed			Ref.	
Employed			0.70 (0.47–1.04)	0.079
Pensioner			0.27 (0.17–0.44)	0.000
Wealth level (quintile)			NA	NA
Poorest	Ref.			
Poor	3.79 (1.86–7.70)	0.000		
Middle	7.89 (3.79–16.4)	0.000		
Rich	2.11 (0.92–4.86)	0.070		
Richest	2.84 (1.27–6.30)	0.010		
Older household members’ comorbidities				
No NCD	Ref.		Ref.	
1 NCD	5.16 (2.27–11.7)	0.000	4.75 (2.00–11.3)	0.000
≥ 2 NCDs	7.05 (3.11–16.0)	0.000	4.72 (1.92–11.6)	0.001
Mean no. outpatient visits by key older household members in the last month				
Primary healthcare facilities	NA	NA	1.55 (1.26–1.89)	0.000
Tertiary health facilities	NA	NA	1.39 (1.13–1.70)	0.002
Private health facilities	2.13 (1.44–3.18)	0.000	1.47 (1.09–1.98)	0.012
Mean no. inpatient admissions for key older household members in the 12 months				
District hospitals	NA	NA	1.72 (1.27–2.34)	0.000
Tertiary hospitals	1.34 (1.06–1.70)	0.015	1.54 (1.27–1.87)	0.000
Urban versus rural			NA	NA
Urban	Ref.			
Rural	2.06 (1.27–3.39)	0.004		
Province				
Tien Giang	Ref.		Ref.	–
Thanh Hoa	5.05 (2.10–12.2)	0.000	1.51 (0.95–2.41)	0.085
Yen Bai	13.7 (5.77–32.4)	0.000	2.00 (1.27–3.17)	0.003
*Results of goodness-of-fit tests* Hosmer–Lemeshow goodness-of-fit test Area under ROC curve	χ^2^ = 5.04, df = 10, *P* = 0.7536 0.9115		χ^2^ = 6.39, df = 8, *P* = 0.6041 0.7831	

“Ref.” indicates the reference group

*CHE* catastrophic health expenditure, *CI* confidence interval, *NA* not applicable, *NCD* noncommunicable disease

The results show that larger households were less likely to suffer CHE (odds ratio [OR] = 0.18) and financial distress (OR = 0.89) than smaller households. Households in which older people had at least one NCD were much more likely to experience CHE and financial distress than households in which older people did not have an NCD (ORs ranging from 4.72 to 7.05). Furthermore, households residing in the mountainous province (Yen Bai) had a higher risk of facing financial catastrophe and distress due to health expenditure than their counterparts in Tien Giang. Households in Thanh Hoa also had a higher likelihood of having CHE than those in Tien Giang. Households headed by pensioners were less likely to suffer financial distress than households in which the head was currently unemployed. Surprisingly, households in the higher wealth quintiles were more likely to face CHE than those in the lowest quintiles (ORs ranging from 2.84 to 7.89). Additionally, rural households were twice as likely to experience CHE as their urban counterparts.

Older people’s health service utilization was strongly correlated with the household’s financial burden resulting from OOPHE. Households in which older adults had more frequent outpatient visits at private health facilities had a higher risk of CHE. Specifically, one unit increase in the average number of outpatient visits at private health facilities by older people doubled the odds of households suffering from CHE (OR = 2.13). In addition, one unit increase in the average number of inpatient admissions by older members at a provincial or central hospital increased the odds of households facing CHE by 1.34 times. Furthermore, all of the variables related to health service utilization were significantly associated with financial distress. For example, a one-unit increase in the average number of outpatient visits by older people at any health facility increased the odds of households suffering from financial distress from 1.39 to 1.55 times. These results indicate that regardless of the type and level of care, the more frequently older household members use health services, the higher the risk that households will encounter financial distress due to health spending.

## Discussion

To the best of our knowledge, this is the first study to examine the magnitude and determinants of financial burdens linked to OOPHE borne by households that include older adults in Viet Nam. The study shows that households that include older people faced CHE and financial distress as economic consequences of OOPHE to provide care for their older members. Furthermore, the demographic and socioeconomic characteristics of households and health service utilization by older people were significant predictors of the financial hardship suffered in this study. The results of this study can contribute a better understanding of whether current financial protection policies for older people are effective, and which older populations are more financially vulnerable and should be targeted for help by future policy interventions.

The study revealed that a considerable proportion of households with older people suffer from CHE. Using the 40% threshold of nonfood household expenditure, the incidence of CHE was 8.6% among households with older people, which was higher than corresponding figures for the general population reported in prior studies in Viet Nam [11, 18]. In a previous study analysing national data collected from 2002 to 2010, the rates of CHE ranged from 3.9% to 5.7% [11]. Notably, the present study found that the health spending for older household members accounted for most of a household’s total expenditure on health, at 86.3%. Older people, especially those with chronic NCDs, require regular healthcare and, consequently, must repeatedly pay for this care [30–32]. Such findings imply that having older people in a household increase the possibility of a household facing financial catastrophe due to OOPHE. This contention is widely supported by evidence from previous studies in Viet Nam [11, 15, 33] and low- and middle-income countries [34–37]. Using data from the VHLSS, two studies concluded that the presence of older adults in a household correlated significantly with higher rates of CHE [11, 33].

Another important finding is that 12.2% of the households facing financial distress due to health expenditures to provide care for older people exceeded their family’s resources. The households that could not pay for services using cash on hand employed coping strategies to finance healthcare costs for their older members, such as borrowing money from others, borrowing from moneylenders or agents [38], and even selling assets [39]. Evidence of such coping mechanisms undertaken by Vietnamese households to deal with unaffordable healthcare costs has been identified in previous studies, such as taking out high-interest loans, selling assets, reducing daily consumption of foods and other necessities, and even forgoing expenses for children's education [15, 38, 40, 41]. Similar to the findings in this study, previous studies in low- and middle-income countries have demonstrated that households with older members and members who have chronic conditions are more likely to use coping strategies to ensure their welfare following the use of health services [42–44]. This study is consistent with other studies showing that borrowing from family or friends and taking out loans are the most common forms of distress financing [19, 39, 45]. In Viet Nam, older people are well connected with their family, friends and communities [32]; thus, social networks seem to be important sources of financial support [46, 47].

Households with older people seem to bear multiple financial burdens associated with their use of healthcare services. First, using a significant proportion of a household’s resources on healthcare for older people may lead households to reduce the family’s consumption of food and other daily necessities and even to a deterioration in living standards [38, 48]. Most older Vietnamese people have not participated in the formal labour force and, consequently, receive limited social welfare benefits [6]. In our study, only one third of older people currently receive retirement benefits. Additionally, because of physical limitations due to ageing, they have less ability to generate income and contribute to the family’s resources [49].

Studies have consistently indicated that, by using these coping strategies to finance their OOPHE, households may successfully address their short-term financial hardship but possibly increase their economic susceptibility in the long run [19, 42, 43, 50]. A previous study by Thuy and colleagues found that Vietnamese households often take out loans to repay current medical debts, which exacerbates their existing financial constraints, making them harder to escape [38]. Similarly, Wagstaff and colleagues found that many households in China take on new debt while already in debt for medical care [51]. Moreover, since many older people cannot care for themselves or independently attend health facilities, other family members have to take time off work to be with them. Consequently, households may bear noticeable indirect costs related to the loss of productivity and income by caregivers through work missed while caring for older household members [12, 38].

In this study, 96.8% of all older adults had health insurance, and in 88.2% of households, everyone had health insurance. One of the crucial objectives of having a health insurance policy is to mitigate the financial consequences of OOPHE, particularly among disadvantaged and vulnerable populations, such as older people, the poor and rural residents [52, 53]. Despite the households sampled having proportionally higher coverage of health insurance than households nationally, the incidence of CHE among households with older people was more significant than that among the general population. Further, the study did not find a significant association between health insurance coverage and financial outcomes for either CHE or financial distress. These findings complement those of earlier studies in Viet Nam, showing that the current health insurance system does not effectively protect households from the financial burden of healthcare spending [11, 15, 54, 55]. As highlighted in a recent study of the population with NCDs, although having health insurance is associated with greater health service utilization, health insurance was not a determinant of CHE and financial distress [15].

These results are in agreement with those observed in prior studies in other countries where health insurance schemes are not well designed and do not protect their beneficiaries from economic hardship [37, 56, 57]. For example, studies have shown that Chinese health insurance programmes had undesirable outcomes and did not protect the insured from financial catastrophic and medical impoverishment, especially the older population [35, 58] and those with NCDs [59, 60]. There are several possible explanations for these findings. First, studies have shown that co-payments for health insurance, even those that are only a small part of the total treatment cost, can cause financial difficulty for patients, particularly those in low-income groups [61, 62]. In some cases, insured patients decided not to use their insurance because of co-payment costs or because medicines covered by their health insurance were not available at the time of service [15]. Second, as argued by Yip and Hsiao, the design of China’s health insurance scheme ignores the type of disease, and the health spending pattern results in a negligible protective effect against CHE [56]. Du and colleagues contended that unfavourable outcomes associated with health insurance schemes might be caused by the high burden of chronic conditions among the older population and their associated health expenditure patterns [63]. Third, limitations on benefits packages that fail to consider the health needs of financially vulnerable groups could partially explain the weak performance of insurance systems [64]. This points to the need to redesign health insurance systems to better protect populations from the economic burden of health expenditures, particularly older populations and disadvantaged groups.

One interesting finding is that health service utilization at tertiary health facilities and private health providers led to higher odds of households with older people suffering CHE. These results reflect our earlier observations among Vietnamese households with members who had NCDs [15]. In Viet Nam, although the same medical procedures or services are provided in both district and provincial hospitals, the fees for services delivered in higher-level facilities are higher than those for the same services offered at the lower levels, and the insured also has higher co-payments for services at higher-level facilities. Recent studies have highlighted gaps in the capacity of primary care health facilities, particularly commune health stations, to provide essential health services for the community owing to factors such as inappropriate financial mechanisms, poor quality and quantity of medicines, lack of equipment and healthcare staff, and a weak health information system [65–68]. These constraints lead to the underutilization of health services at primary care facilities, where insured patients are less likely to incur co-payments and additional out-of-pocket spending. Weraphong and colleagues suggested that bypassing or underutilization of designated primary care facilities can lead insured patients to use private care and public providers outside their local areas, increasing their nonmedical and indirect costs and potentially pushing households into financial catastrophe and impoverishment [69]. Moreover, the current study found that, regardless of the type or level of care, health service utilization by older household members resulted in a higher possibility of a household encountering financial distress related to spending on healthcare. Given that almost all older people in our sample were insured, this association is notable but not surprising, because the weak financial protection function of Viet Nam's health insurance system has been broadly discussed [11, 15, 54, 55].

Multivariate regression analyses showed significant associations between key household characteristics and the financial burden of health expenditures. This study indicates that smaller households are more likely to suffer CHE and financial distress than households with more members. One possible explanation for this is that households with only older people or fewer members have fewer people to contribute income and, consequently, had limited financial resources; thus, they may be at higher risk of financial hardship [36]. Notably, nearly half of the households in this study consisted of only older people living independently or with their spouses. This finding aligns with those observed in China and Nigeria [36, 70, 71], and also in Viet Nam [11, 72]. In China, several studies consistently found that empty-nest households consisting of older people living alone had greater odds of incurring CHE than those in which an older person lived with a spouse or in a multigenerational household [70, 71]. These findings might further suggest the need to implement a financial protection policy that can lower the risk of the financial burden for households with only older people.

This study confirms the high prevalence of NCDs among the older population, as found in previous studies in Viet Nam [6, 12]. The presence of older people with NCDs in a household was a strong predictor of CHE and financial distress. Furthermore, households where older people had NCDs were seven times as likely to incur CHE as households in which older people had no NCDs. These findings broadly support the work of other studies and highlight the economic burden of NCDs on households [44, 73] and particularly among older households [12, 35, 74]. Patients with chronic NCDs require more frequent health services; therefore, they are more likely to incur OOPHE than those without NCDs [12, 15, 75]. In a multicountry study, Lee and colleagues demonstrated that NCD multimorbidity is highly correlated with more regular use of health services and a higher economic burden for the population [75]. As suggested in the literature, households in which older members with NCDs seek regular care also incur more direct nonmedical costs (e.g. transportation, food and accommodation for patients and caregivers) [76–78] and indirect costs (e.g. income loss as caregivers take time off from work) [12, 38]. The findings of this study strengthen the evidence for the poor performance of the existing health financing system in protecting households from the negative economic impact of NCDs, particularly those households with older people.

The current study also found that older households in rural areas or Yen Bai (a remote, mountainous province) are more likely to incur CHE than those living in urban and lowland areas (e.g. Tien Giang). These results are in keeping with those of Minh and colleagues, who found, through a secondary analysis of 10 years of national data, that living in a rural area was linked with a higher risk of CHE [11]. Similar findings have been reported from other countries [79], such as China [58] and India [73]. This implies that households with older people living in low-resource settings are more financially vulnerable than their counterparts residing in more socioeconomically advantaged areas. This further highlights the need for policies that more effectively target households with older people living in rural and remote, mountainous areas, because these households are at greater risk of suffering financial catastrophe due to healthcare use.

It is somewhat surprising that older households in the higher wealth quintiles are more likely to face CHE than those in the lowest quintile. This is contrary to prior studies in other countries demonstrating that low-income households with older people have a higher risk of financial hardship due to health expenditure than higher-income households [35, 36, 58]. Several factors could explain this observation. First, poor households may not spend much out of pocket due to limited resources, while better-off households spend more on health services. Likewise, other studies have reported that wealthier households incur more OOPHE than poorer households [37, 80]. Second, there is evidence that poor people, even those with health insurance, do not access institutional care because of the health insurance co-payment and associated nonmedical and indirect costs. Instead, they may resort to self-medication or forgo treatment because of the expected costs; however, this may cause more severe health complications and lead to further financial difficulties [19, 81]. The data from our study show that poor households used self-medication more often and less outpatient and inpatient care than higher-quintile households. Therefore, a further qualitative investigation is needed to understand older people's healthcare-seeking behaviour and coping mechanisms in response to the need to pay for healthcare.

Finally, several important limitations of this study need to be acknowledged. First, this study used self-reported data on health service use, OOPHE and NCDs; thus, the data are subject to recall bias. Second, the cross-sectional design limits the findings, making it impossible for us to draw causal interpretations of the associations. Given that Viet Nam has plans to improve financial protection for its population, further longitudinal studies may help in assessing the causal impact of such policy interventions on health service utilization and financial outcomes among the targeted populations. Fourth, several studies indicate that NCD comorbidities are prevalent among the younger population in Viet Nam [82, 83]; however, the multivariable regression models could not adjust the presence of NCD among younger household members, which could affect households’ financial outcomes. Finally, the study collected information about OOPHE using disaggregated items by illness episode, while aggregated items about OOPHE were used to collect data about health spending for other family members. Evidence has indicated that a lower degree of disaggregation of out-of-pocket items could underestimate the mean OOPHE [84].

## Conclusions

In response to the increased need for healthcare among older people, the government of Viet Nam has been gradually developing policies to improve older people's access to health services and protect them from financial consequences linked to health service utilization. Although the limited impact of existing financial protection policies in Viet Nam has been widely discussed in the literature, the current study appears to be the first to investigate the financial burden of OOPHE on the older population and their households. The findings provide critical confirmation that Vietnamese households are more likely to experience CHE and financial distress due to expenditures for healthcare for older household members.

The results of the regression analyses identified certain factors that make these households more financially vulnerable than others. In particular, households are at a higher risk of incurring financial burdens related to health expenditures if they have a smaller size and include only older people; are in rural or remote, mountainous areas; and have older household members with NCDs. Consistent with prior studies in Viet Nam, this study did not find a meaningful relationship between having health insurance and financial burden. Furthermore, having older household members who sought care at private providers and more specialized hospitals increased the possibility of a household facing CHE. Regardless of the type and level of care, health service utilization by older people results in a higher likelihood that a household will experience financial distress related to spending on health. Our findings show that the current social health insurance scheme, which aims to protect beneficiaries against financial difficulties, seems not to have achieved its policy objective, particularly for older people.

The findings of this study have several important implications for policy development. International experience suggests that the implementation of health insurance alone will not directly decrease OOPHE: reform also needs to be undertaken on the health financing mechanisms linked to the health insurance system [85]. Redesigning benefit packages to target and prioritize the needs of older people is recommended, particularly aiming to help those with chronic NCDs and those in disadvantaged groups, such as poor people and those living in rural and remote areas. For example, the health insurance programme might consider waiving co-payments for older adults with chronic conditions who require long-term treatment with medicines. Further research could examine the distribution patterns of the costs of health services for older people as an initial step towards developing a comprehensive and financially protective service package for older adults. Another possible strategy could involve reforming provider payment mechanisms to control overprescribing and service abuse while enhancing primary healthcare services and discouraging more expensive hospital-based care. Finally, it is also strongly recommended that primary healthcare facilities be strengthened so that care can be provided closer to home at lower costs, particularly for the management of NCDs.

## Supplementary Information

**Additional file 1.** Appendices.

## Data Availability

The data that support the findings of this study are available from the Health Strategy and Policy Institute; however, restrictions apply to the availability of these data, which were used under license for the current study, so they are not publicly available. However, data are available from the authors upon reasonable request and with permission of the Health Strategy and Policy Institute.

## Abbreviations

OOPHE: Out-of-pocket health expenditure
CHE: Catastrophic health expenditure
NCD: Noncommunicable disease
OR: Odds ratio
