# Supplementary material for: Household financial burden associated with healthcare for older people in Viet Nam: a cross-sectional survey
Source: Health Res Policy Syst. 2022 Nov 29;20(Suppl 1):112. doi: 10.1186/s12961-022-00913-3 (PMC9706832; doi:10.1186/s12961-022-00913-3)
Supplement: Supplementary file 1 — Additional file 1. Appendices. [file 12961_2022_913_MOESM1_ESM.docx]

**APPENDICES**

**Appendix 1. Average out-of-pocket spending per household for interviewed older people (unit: thousand VND)**

|  | **No. households with older people using health services** | **Co-payment** | **Direct medical exp** | **Direct non-medical exp** | **Other non-medical exp** |
| --- | --- | --- | --- | --- | --- |
| **Self-medication (last 4 weeks)** | | | | | |
| Thanh Hoa | 228 |  | 585.6  (0-6,500) |  | |
| Tien Giang | 79 |  | 483.0  (0-13,880) |  |  |
| Yen Bai | 116 |  | 826.2  (0-7,690) |  |  |
| Urban | 196 |  | 791.9  (0-13,880) |  |  |
| Rural | 227 |  | 494.7  (0-7,600) |  |  |
| Multi-generational households | 226 |  | 522.2  (0-7,600) |  |  |
| Older households | 197 |  | 758.8  (0-13,880) |  |  |
| **Outpatient care (last 4 weeks)** | | | | | |
| Thanh Hoa | 177 | 32.9  (0-2,500) | 364.8  (0-10,000) | 20.3  (0-950) | 2.1  (0-250) |
| Tien Giang | 311 | 47.3  (0-2,000) | 507.9  (0-8,500) | 87.0  (0-3,000) | 51.4  (0-7,300) |
| Yen Bai | 317 | 31.1  (0-2,200) | 467.0  (0-21,300) | 35.3  (0-1,000) | 20.9  (0-3,000) |
| Urban | 431 | 34.7  (0-2,200) | 437.0  (0-21,300) | 38.4  (0-1,000) | 47.0  (0-7,300) |
| Rural | 374 | 41.3  (0-2,500) | 487.2  (0-10,000) | 67.7  (0-3,000) | 7.2  (0-600) |
| Multi-generational households | 466 | 35.9  (0-2,500) | 443.7  (0-21,300) | 44.5  (0-1,500) | 14,0  (0-3,000) |
| Older households | 339 | 40.3  (0-2,200) | 483.2  (0-10,000) | 62.3  (0-3,000) | 48,4  (0-7,300) |
| **Inpatient care (last 12 months)** | | | | | |
| Thanh Hoa | **108** | 1788.1  (0-105,000) | 1158.3  (0-18,000) | 818.7  (0-17,000) | 111.6  (0-4,700) |
| Tien Giang | 61 | 1932.1  (0-35,000) | 1247.5  (0-31,600) | 1277.2  (0-32,000) | 1279.3  (0-36,800) |
| Yen Bai | 100 | 472.0  (0-10,000) | 2179.6  (0-22,500) | 915.1  (0-12,000) | 170.5  (0-10,000) |
| Urban | 141 | 1874.4  (0-105,000) | 1924.0  (0-31,600) | 1136.5  (0-32,000) | 693.9  (0-36,800) |
| Rural | 128 | 733.5  (0-45,000) | 1155.2  (0-22,500) | 762.4  (0-17,000) | 72.6  (0-6,000) |
| Multi-generational households | 148 | 666.4  (0-32,000) | 1554.3  (0-22,500) | 849.1  (0-17,000) | 398.6  (0-15,000) |
| Older households | 121 | 2145.0  (0-105,000) | 1563.0  (0-31,600) | 1092.4  (0-32,000) | 397.9  (0-36,800) |
| **Long-term care (last 12 months)** | | | | | |
| Thanh Hoa | 19 |  | | 5583.2  (0-48,000) |  |
| Tien Giang | 13 |  |  | 1336.2  (0-7,800) |  |
| Yen Bai | 23 |  |  | 26020.9  (0-162,500) |  |
| Urban | 30 |  |  | 14053.3  (0-162,500) |  |
| Rural | 25 |  |  | 12013.2  (0-78,000) |  |
| Multi-generational households | 25 |  |  | 7554.8  (0-48,000) |  |
| Older households | 30 |  |  | 17768,7  (0-162,500) |  |

Note: out-of-pocket spending for health includes health insurance co-payments, direct medical expenses (e.g. medications, laboratory tests, consultation fees), direct non-medical expenses associated with accessing health services (e.g. food, transportation, and accommodation), and other non-medical expenses (e.g. informal gifts for health providers).

**Appendix 2. Model buildings process with the applications of backward elimination approach**

As the outcomes of interest were binary variables, multivariable binary logistic regression analysis was employed to examine the factors associated with the catastrophic health expenditure (yes/no) and financial distress (yes/no). We used the backward elimination method to determine the final models as follows

1. Initially, bivariate analysis was conducted to explore the potential factors associated with the outcomes, which informs the variables included in the base multivariate regression models. The likelihood-ratio (LR) test was used in both univariate and multivariate models to test the effect of independent variables in the regression model. As a rule of thumb, the significant level of variables in univariate models used for considering the inclusion of an explanatory variable in the base model is p<0.25.
2. **Retaining significant variables and assessing confounding and interaction effect.**

- We removed one variable at a time from the base model. The new model that removed the variable was tested against the full one using a likelihood ratio test. If the p-value of the likelihood ratio test is greater than 0.05, the variable could be dropped from the model. At each round, we removed the insignificant variable (p-value > 0.05) with the highest p-value. This procedure continued until all remaining variables made a significant contribution to the model (P <= 0.05).
- Given that some potential confounders were identified, the effect modification of each was examined by including interaction terms one at a time to the model created by multiplying the study variable and the potential confounder. A likelihood ratio test assessed the significance of the interaction terms. As a common practice, interaction terms are retained in the model only if they are significant at p<0.01. The magnitude of the confounding of a variable is evaluated by the change in the value of the estimated effect of when the potential confounder is removed from the model. If the exclusion of a variable makes a significant change in the model, as a rule of thumb greater than 10% change of the regression coefficients of any remaining variables, it would be considered a confounder and eliminated from the model. However, if including the potential confounding factor in the model reduces the standard error of the other variables to a significant extent, it is retained in the model. If the variable is not statistically significant in the model but alters the associations of interest appreciably, it is retained in the model.
- The analysis identified the variables causing the effect modification in the associations of interest. We give an example with the association of health insurance status and the outcome variable. First, the variables or factors that could be confounders in the association between the study variable and the outcomes were determined. Two key criteria for identifying the confounder are (i) the variable must be associated with the outcome factor in the population, and (ii) the variable must also be related to the study variable in the data. For the first criterion, the information on the associations was obtained from the literature. The inclusion of the mentioned independent variables was based on evidence found from the comprehensive literature on this topic. Regarding the second criterion, the association between insurance status and other individual independent variables were examined using either the Chi-square test (for categorical variables) or the T-test (for continuous variables). A significant association between an independent variable and a study factor may indicate that this variable could be a confounding factor.

1. **Model diagnostic:** the most common method to examine whether a logistic regression model fits well with the data is the Hosmer-Lemeshow test. We divided the data set into ten subgroups because of having the continuous predictor variable. The observed and expected frequencies in each group are calculated to conduct a Chi-squared test with 2 degrees of freedom. The null hypothesis for the Hosmer-Lemeshow test is that there is a goodness of fit between the model and the data; thus, a non-significant test result (P > 0.05) indicates a good fit. Moreover, to identify whether the fitted regression model could discriminate between the observed and predicted frequency, the degree of matching was evaluated by employing a measure called the area under the Receiver Operating Characteristics (ROC) curve.
2. The variance inflation factor was used to **check multicollinearity**, and a factor of <10 was considered acceptable

**Appendix 3. Base logistic regression models predicting the determinants catastrophic health expenditure and financial distress among households with people ≥60 years, Viet Nam**

| **Variable** | **Catastrophic health expenditure** (WHO’s definition) | | **Financial distress** | |
| --- | --- | --- | --- | --- |
|  | **Unadjusted OR**  **(95% CI)** | ***P*** | **Unadjusted OR (95% CI)** | ***P*** |
| Household size | 0.67 (0.11 - 0.25) | 0.000 | 0.90 (0.81 – 1.00) | 0.051 |
| Household head aged ≥60 years  No  Yes | ref  0.76 (0.20 – 2.90) | 0.689 | NA | NA |
| Educational level |  |  |  |  |
| Elementary or less | ref |  | ref |  |
| Secondary school | 1.57 (0.84 – 2.92) | 0.158 | 1.10 (0.71 – 1.72) | 0.663 |
| High school or higher | 1.76 (0.89 – 3.51) | 0.105 | 1.03 (0.62 – 1.71) | 0.624 |
| Head of household’s occupational status | ref  1.04 (0.57 – 1.90)  0.64 (0.34 – 1.20) | 0.900  0.162 |  |  |
| Unemployed |  |  | 0.46 (0.27 – 0.80) | 0.006 |
| Employed |  |  | ref |  |
| Pensioner |  |  | 1.48 (0.98 – 2.42) | 0.061 |
| Wealth level (quintile) |  |  | ref  1.09 (0.65 – 1.82)  1.04 (0.60 – 1.79)  0.68 (0.36 – 1.27)  0.83 (-.43 – 1.59) | 0.740  0.888  0.227  0.570 |
| Poorest | ref |  |  |  |
| Poor | 3.62 (1.72 – 7.63) | 0.001 |  |  |
| Middle | 8.06 (3.69 – 17.6) | 0.000 |  |  |
| Rich | 2.15 (0.88 – 5.23) | 0.092 |  |  |
| Richest | 2.80 (1.16 – 6.75) | 0.022 |  |  |
| Older household members’ comorbidities |  |  |  |  |
| No NCD | ref |  | ref |  |
| 1 NCD | 5.31 (2.30 – 12.3) | 0.000 | 4.81 (2.02 – 11.5) | 0.000 |
| ≥2 NCDs | 6.65 (2.83 – 15.6) | 0.000 | 4.70 (1.91 – 11.6) | 0.001 |
| Mean no. monthly outpatient visits by older household members | | | | |
| Primary health care facilities | NA |  | 1.56 (1.27 – 1.91) | 0.000 |
| Tertiary health facilities | 1.19 (0.92 – 1.54) | 0.179 | 1.40 (1.13 – 1. 72) | 0.002 |
| Private health facilities | 2.23 (1.50 – 3.32) | 0.000 | 1.52 (1.12 – 2.06) | 0.006 |
| Mean no. inpatient admissions for older household members (previous 12 months) | | | | |
| District hospitals | 1.09 (0.68 – 1.73) | 0.722 | 1.72 (1.26 – 2.34) | 0.001 |
| Tertiary hospitals | 1.33 (1.04 – 1.69) | 0.019 | 1.54 (1.27 – 1.87) | 0.000 |
| Health insurance status |  |  |  |  |
| Not fully insured | ref |  | NA |  |
| Fully insured | 1.15 (0.35 – 3.79) | 0.815 |  |  |
| Urban versus rural |  |  | ref  1.08 (0.73 – 1.59) | 0.690 |
| Urban | ref |  |  |  |
| Rural | 1.91 (1.14 – 3.20) | 0.014 |  |  |
| Province |  |  |  |  |
| Tien Giang | ref | 0.000 | 0.65 (0.39 – 1.08) | 0.095 |
| Thanh Hoa | 5.07 (2.04 – 12.6) |  | ref |  |
| Yen Bai | 13.5 (5.44 – 33.4) | 0.000 | 1.28 (0.82 – 1.98) | 0.279 |

“ref” indicates the reference group.

CI: confidence interval; NA: not applicable; NCD: noncommunicable disease.
